# Understanding barriers to treatment regimen adherence in Pakistani women with polycystic ovary syndrome: a cross sectional study

**DOI:** 10.4314/ahs.v26i2.17

**Published:** 2026-06

**Authors:** Moeza Zulfiqar, Waqas Akram, Muhammad Talha, Muhammad Zulnurain, Emaan Saif, Ali Akhtar

**Affiliations:** Faculty of Pharmaceutical Sciences, University of Central Punjab, Lahore, Pakistan

**Keywords:** Polycystic Ovary Syndrome, Treatment adherence, Barriers, Socioeconomic factors, Pakistan

## Abstract

**Background:**

Polycystic ovary syndrome is a prevalent endocrine disorder among reproductive-age women, significantly impacting their quality of life and increasing the risk of chronic diseases.

**Objectives:**

This study aimed to determine adherence levels and main barriers to adherence to prescribed treatment regimens among women with PCOS in Lahore, Pakistan.

**Methods:**

A cross-sectional study was conducted at hospitals (government and private) in Lahore, Pakistan, enrolling 401 women diagnosed with PCOS. Data were collected using structured questionnaires.

**Results:**

Only 21.4% of participants reported full adherence to their treatment regimens, while 50.6% were partially adherent, and 27.9% were non-adherent. Significant associations were found between adherence and socioeconomic status (SES) (χ^2^ = 8.98, p = 0.030) and body mass index (BMI) (χ2 = 8.28, p = 0.041). Logistic regression revealed women from upper-middle-class backgrounds had significantly lower odds of adherence compared to upper-class women (OR = 0.55, 95% CI: 0.33–0.92; p = 0.022).

**Conclusion:**

Treatment adherence among women with PCOS in Pakistan is suboptimal. Socioeconomic status and body mass index were significant predictors of adherence, while major barriers included limited knowledge, medication side effects, and treatment cost. Targeted interventions involving patient education, socioeconomic support, and healthcare system improvements are necessary to enhance treatment adherence and outcomes in this population.

## Introduction

Polycystic Ovarian Syndrome (PCOS) is one of the most prevalent endocrine disorders affecting the women of reproductive age. Based on prevalence estimates, PCOS affects 6%–18% of women worldwide 1. Among Pakistani women, a surprisingly high prevalence of 52% has been documented 2.

PCOS is diagnosed when at least two of the three Rotterdam criteria are met, and it is characterized by a range of distinct phenotypes:(i) hirsutism, acne, seborrhea, and alopecia accompanied by clinical hyperandrogenism and/or high levels of circulating testosterone; (ii) ultrasound examination to determine whether ovarian cysts are present; and (iii) oligo-amenorrhea with oligo-anovulation 3. Pharmacological Treatment options commonly prescribed for managing PCOS include Metformin, Oral Contraceptive Pills, and Anti-androgens. Additionally medications like thiazolidinediones (TZDs), glucagon-like peptide-1 (GLP-1) receptor agonists, insulin-sensitizing agents, aromatase inhibitors (AIs), and antioxidants may also be used 4. Among all, Lifestyle modifications form the cornerstone of PCOS management 5.

Adherence to prescribed medication regimen is an important factor to achieve optimal therapeutic benefits. Non-adherence to treatment results in severe complications such as hyperlipidemia, cardiovascular diseases, gestational diabetes, preeclampsia, and infertility 1. Therefore, long-term adherence to recommended medications and lifestyle modifications are necessary for the optimal management of PCOS and reduction of clinical problems. Studies indicate that adherence to lifestyle modifications, such as diet and physical activity, is often challenging for patients but remains crucial for achieving expected treatment outcomes 6.

In South Asia, sociocultural norms, limited health literacy, and inadequate access to specialized care further exacerbate these challenges. An Indian study by1 highlighted that barriers such as insufficient knowledge, treatment burden, and lifestyle challenges significantly hinder adherence. However, no study to date has comprehensively explored these barriers within Pakistan, where the healthcare infrastructure, socioeconomic conditions, and cultural attitudes toward women's reproductive health differ markedly. According to an Iranian study, the most common obstacles were lengthy treatment courses, many follow-up appointments, and side effects like anorexia, lethargy, hot flushes, and mastalgia 7.

Despite increasing prevalence of PCOS in a developing country like Pakistan 8, little is known about the specific barriers to treatment adherence in this region. Pakistani women often rely on family members for healthcare decisions, face limited opportunities for physical exercise due to social restrictions, and encounter financial and accessibility constraints in long-term disease management. These contextual differences underscore the importance of localized evidence to design culturally sensitive and feasible interventions. Therefore, the present study aimed to assess the level of treatment adherence, identify barriers to adherence, and determine sociodemographic predictors influencing adherence among women with PCOS in Lahore, Pakistan. By addressing this gap, the findings are expected to inform healthcare providers and policymakers in developing tailored strategies to enhance treatment adherence and improve women's health outcomes in similar settings.

## Methodology

### Study Design

This study used a cross-sectional design to assess treatment adherence among Pakistani women with PCOS. Data collection was carried out over a period of four months from December 2024 to March 2025.

### Study Setting

The study was conducted in both government and private healthcare settings within Lahore, Pakistan. Participants were recruited from five major tertiary care hospitals. These hospitals were selected to ensure a representative sample, encompassing diverse patient populations and varied healthcare delivery practices across different socioeconomic backgrounds.

### Study Population

The study included women diagnosed with PCOS aged between 13 and 45 years. Women were selected based on the following inclusion criteria:

- Inclusion Criteria: Women aged 13-45 years, diagnosed with PCOS based on the Rotterdam criteria.
- Exclusion Criteria: Exclusion criteria included women with comorbidities that could confound adherence assessment, such as thyroid disorders, diabetes mellitus, Cushing's syndrome, major psychiatric illness, or other chronic endocrine disorders. Pregnant or lactating women were also excluded.

Patients attending the gynecology and endocrinology outpatient departments were initially identified, and snowball referrals were used to include additional participants meeting eligibility criteria. This hybrid approach was chosen due to the absence of a centralized PCOS registry in Pakistan and to ensure adequate representation across hospital settings. This approach ensured a varied sample and allowed for the inclusion of women from both urban and rural backgrounds in Lahore.

### Sample Size

The sample size for the study was calculated using Daniel's equation, taking into account a 20% dropout ratio. The final sample size was determined to be 401 participants, which was sufficient to ensure statistically significant results and adequate power for the analyses.

### Data Collection

Data was collected using a structured questionnaire adapted from previously published study 9. The tools used for data collection were demographic proforma, with seven items on baseline characteristics and six items on treatment plan, a barrier assessment questionnaire consisting of 19 items. The tool assessed barriers such as knowledge gaps, financial burden, medication side effects, and lifestyle challenges. It was pilot-tested on 30 participants for comprehension and reliability, yielding a Cronbach's α of 0.79. The MARS scale demonstrated acceptable internal consistency in this study (Cronbach's α = 0.82).

It was administered by the researcher to participants who were unable to read or understand English. For those who were able to read and understand English, the questionnaire was self-administered.

The questionnaire covered the following areas:

- Demographics: Age, marital status, education, occupation, socioeconomic status, BMI, etc.
- Treatment Adherence: Adherence to medication, lifestyle modifications, and other treatment regimens.
- Barriers to Adherence: Socioeconomic factors, cultural beliefs, healthcare system-related issues, medication side effects, and lack of knowledge.

Operational Definitions

- Socioeconomic Status (SES): Classified into four categories (upper, upper-middle, lower-middle, and lower) based on self-reported monthly household income and occupation, following national income classification standards 10.
- Adherence Levels: Determined using total MARS scores. Participants scoring in the upper tertile were categorized as highly adherent, middle tertile as partially adherent, and lower tertile as non-adherent. For regression analysis, adherence was dichotomized into adherent (high) and non-adherent (partial + low) categories.
- Body Mass Index (BMI): Categorized according to WHO criteria (underweight <18.5, normal 18.5–24.9, overweight 25–29.9, obese ≥30 kg/m^2^).

### Statistical Analysis

Data analysis was performed using SPSS software (version 26). Descriptive statistics were presented as frequencies and percentages for categorical variables and means with standard deviations for continuous variables. To identify associations between two categorical variables Pearson's chi-square tests were used, between categorical and continuous variables independent sample t-tests were used where there were two categories. Binary logistic regression was conducted to identify significant predictors of adherence, with odds ratios (ORs), 95% confidence intervals (CIs), and p-values reported. The level of statistical significance was set at p < 0.05.

## Ethical Considerations

Ethical approval was obtained from the Institutional Research Board (IRB) and the Institutional Ethical Committee of University of Central Punjab, Lahore ensuring adherence to ethical standards and research protocols. Informed consent was obtained from all participants, ensuring that they understood the study's purpose and procedures. All participant data was kept confidential, with personal identifiers such as names removed to maintain anonymity.

## Results

### Participants Characteristics

A total of 401 women diagnosed with polycystic ovarian syndrome (PCOS) participated in the study. The mean age of participants was 27.8 ± 6.2 years. Most were aged between 21 and 28 years (48.4%), and a majority (57.9%) were single. Educationally, approximately 44.9% had completed undergraduate studies, and occupationally, nearly half (48.1%) were homemakers. Socioeconomically, the largest group belonged to the upper-middle class (32.4%). Urban residents formed the majority at 54.9%. In terms of BMI, 42.6% of participants were of normal weight, followed by 37.2% overweight individuals.

### Prescribed Treatment Regimens

Metformin was the most commonly prescribed medication (32.7%), with oral contraceptive pills used by 25.7% of participants. Anti-androgens (20.2%) and other hormonal therapies (21.4%) were also reported. Physical activity recommendations were frequently reported, notably brisk walking (42.6%). Diet-related changes were popular, though junk food consumption was reported by 37.9%. Adherence levels indicated 21.4% fully adherent, 50.6% partially adherent, and 27.9% non-adherent to prescribed treatments.

Among the 401 participants, only 21.4% were fully adherent to their treatment regimen, while 50.6% were partially adherent, and 27.9% were non-adherent. Partial Adherence emerged as the most common pattern observed.

### Barriers to Treatment Adherence

Primary personal barriers included lack of time for exercise (66.3%) and exercise being perceived as tiring (58.6%). Treatment-related barriers notably included long therapy duration (62.3%) and medication side effects (54.4%), contributed to poor adherence. Healthcare system-related barriers such as long distances to hospitals (45.9%), difficulty in getting appointments (42.1%) and insufficient communication by healthcare providers (39.7%) also significantly impacted adherence.

### Association Between Demographic Variables and Adherence (Using Chi-Square Test)

Chi-square tests revealed a statistically significant association between adherence and socioeconomic status (χ^2^(3) = 8.980, p = 0.030) and between adherence and BMI category (χ2(3) = 8.278, p = 0.041). No significant associations were found between adherence and age group, marital status, education level, socioeconomic status, or residence.

### Predictors of Adherence

Binary logistic regression was performed to identify sociodemographic predictors of adherence among women with PCOS. The final model showed statistical significance for certain variables (χ2 = 17.125, df = 6, p = 0.009), with a Nagelkerke R2 of 0.056, suggesting the model explained about 5.6% of the variance in adherence behaviour.

Among all predictors, socioeconomic status and BMI were significantly associated with treatment adherence. Participants from upper-class backgrounds had significantly higher odds of adherence compared to those from lower-class backgrounds (OR = 1.115; 95% CI: 0.328–0.918; p = 0.022). Similarly, among BMI categories, normal-weight women had higher odds of adherence compared to those categorized as obese (OR = 1.467; 95% CI: 0.704–3.056; p = 0.048).

Other variables such as age group, marital status, education, occupation, and residence did not show significant associations with adherence (p > 0.05). These results suggest that adherence is more likely among women with higher socioeconomic stability and healthier BMI levels.

## Discussion

In this study, we found suboptimal adherence to prescribed treatment regimens. Only a minority of participants achieved full adherence to both medication and recommended lifestyle modifications, while a substantial proportion were partially adherent or completely non-adherent. These findings echo the concern that PCOS patients often struggle with long-term treatment engagement, consistent with reports from other populations where adherence rates ranged widely (as low as ~22% in some studies) 11. The low overall adherence observed is alarming, as insufficient adherence can exacerbate PCOS symptoms and heighten risks of complications like infertility, metabolic disorders, and poor quality of life 9. It underscores that despite the availability of effective therapies for PCOS, their real-world impact is limited by patients' ability and willingness to follow through with treatment plans. The relatively lower adherence observed in this study compared to the Indian cohort (21.4% vs. 32%) highlights the potential influence of contextual factors, including differences in literacy rates, healthcare access, and cultural attitudes toward women's reproductive health in Pakistan.

An understanding of the barriers faced by these women helps interpret our results. Participants reported a range of personal, treatment-related, and system-level obstacles impeding adherence. Common personal barriers included low motivation for prolonged lifestyle changes, and misconceptions about PCOS. Notably, many women lacked adequate knowledge about the disease and its management, leading to poor self-efficacy in following recommendations – a trend also identified in other studies 9, 12. Treatment-related barriers were also prevalent: side effects of medications (e.g., gastrointestinal upset with metformin) and the perceived lack of immediate symptom relief were cited as reasons for discontinuing therapy. The long duration of treatment required for PCOS (often lifelong lifestyle changes and months of pharmacotherapy) further contributed to decreasing adherence, as patients grew impatient for results 9. Additionally, difficulties in adhering to prescribed diets (reports of “bland” or culturally incompatible diets) and exercise regimens were noted, reflecting the challenge of sustained lifestyle modification. Healthcare system barriers played a role as well – for instance, cost of medications or consultations, limited access to specialized care, and fragmented follow-up. Some women described short, rushed consultations and insufficient counselling by healthcare providers, leaving them poorly informed about the importance of continuing treatment. This aligns with known issues in PCOS care where patients often do not receive adequate education on long-term risks 13. Collectively, these barriers explain the moderate-to-low adherence levels: even women who start therapy may falter over time without sufficient support and understanding. Our analysis also identified important predictors of adherence. Socioeconomic status (SES) emerged as a significant factor: women from higher SES backgrounds tended to have better adherence rates compared to those from lower SES. This may be because higher SES often translates to better education, health literacy, and financial ability to afford treatments and healthy foods. Indeed, prior research in South Asia found that women with higher SES were more likely to understand the consequences of non-adherence and had resources to follow treatment advice 9. In contrast, lower SES women in our study faced compounded challenges – for example, difficulty paying for medicines or gym memberships, less flexible work/life schedules for medical visits, and possibly lower baseline knowledge about PCOS. These financial barriers are particularly relevant in Pakistan, where out-of-pocket expenditure accounts for more than 60% of total healthcare spending, disproportionately affecting women from lower socioeconomic backgrounds. The other key predictor was BMI: we found that women with higher BMI (indicative of overweight/obesity) were less adherent, particularly to lifestyle interventions. This mirrors observations in other populations – a study from China reported that a patient's BMI significantly affected adherence, with obesity linked to poorer compliance 14. Higher-BMI patients may encounter more physical discomfort and psychological barriers when attempting exercise or dietary change, leading to frustration and dropout. It is also possible that obese women with PCOS experience more severe symptoms (e.g. fatigue, depression) that undermine their motivation to stick with treatment. On the other hand, normal-weight women might find it relatively easier to incorporate lifestyle advice or may feel more immediate benefits, aiding adherence. Our finding that BMI and SES influence adherence highlights how health behaviours are intertwined with social determinants – an important consideration for tailoring interventions.

### Comparison with Global and Regional Literature

Our results are largely congruent with findings from other settings, although some differences emerge in comparison. The adherence rates we observed fall in line with those reported in similar low- and middle-income country contexts. For example, an Indian study of 224 women with PCOS found that only about one-third (32.1%) were fully adherent to treatment, with the remainder partially or non-adherent 9. This is strikingly similar to our cohort's adherence distribution, suggesting that poor adherence is a common problem in South Asian populations. The lower adherence in Pakistani women may also stem from lower health literacy, stigma associated with reproductive disorders, and fewer specialized clinics providing PCOS counseling. Unlike in India, where urban health awareness programs are more widespread, Pakistan lacks structured community-level interventions addressing PCOS. Another study from Kashmir (northern South Asia) reported slightly higher medication adherence (around 41% high adherence) but still a majority with suboptimal compliance 15, though that study did not assess lifestyle adherence which could explain the somewhat better rates.

Globally, a systematic review by 11 noted wide variation in adherence to PCOS treatments (ranging ~22% to 86%), but importantly, many studies clustered at the lower end of that range 11. Our findings align with the lower adherence figures commonly seen in routine practice, underscoring that Pakistan is not an outlier; rather, it reflects the general challenge of managing a chronic condition like PCOS that requires long-term behaviour change.

When examining barriers to adherence, our study's qualitative findings closely mirror those documented elsewhere, affirming some universal themes. Lack of knowledge and understanding about PCOS was one of the top barriers for our participants, and this has been echoed in diverse contexts. In India, studies found that a majority of women had insufficient knowledge of the disease and its management as a primary barrier to compliance 9. Similarly, in an Iranian qualitative study, women attributed non-adherence partly to inadequate information – both their own and among family members – and insufficient explanation from doctors 12. This convergence suggests that patient education gaps are a pervasive issue transcending borders. Side effects and dissatisfaction with treatment outcomes also came up frequently across studies. Indian women commonly reported stopping medications due to side effects or perceiving “no relief of symptoms” after some time 9. In Iran, side effects (like lethargy, hot flashes, etc.) and the long duration of therapy were noted barriers as well 9. Our participants voiced analogous frustrations, often abandoning therapies when immediate improvements were not evident. This pattern highlights a psychological aspect of adherence: chronic conditions like PCOS may not have the quick feedback loop that patients hope for, leading to impatience. In terms of lifestyle adherence, challenges such as maintaining a prescribed diet or exercise regimen are well documented. An Indian and a Singaporean study both observed that women struggled with dietary advice, especially when it conflicted with local food preferences or when healthy options were expensive 9,16. We similarly found dietary non-compliance to be common, reflecting how cultural food habits (e.g., high-carbohydrate South Asian cuisine) and the effort needed for meal planning can impede adherence to recommended diets.

Despite these similarities, there are some notable differences in emphasis, likely stemming from socio-cultural and healthcare system variations. For instance, family and social influences appeared particularly salient in our context and some regional studies. Many Pakistani women live in joint families or rely on family support for healthcare decisions; accordingly, lack of family support was cited as a barrier (e.g. husbands or parents not prioritizing the woman's treatment). This is in line with reports from Iran and India: an Iranian study noted that friends or family sometimes actively discouraged women from seeking care, deeming the diagnostic procedures too painful or the treatments too costly 12. Such social discouragement can be rooted in cultural norms that trivialize menstrual or hormonal issues as “ordinary” or fear of medical expenses. In contrast, Western studies rarely mention familial opposition as an adherence barrier, reflecting different family dynamics. Instead, studies from high-income countries emphasize barriers like time constraints (busy work schedules making it hard to exercise or attend appointments) and financial costs of healthy lifestyle choices or fertility treatments 17. Interestingly, our participants – many of whom were homemakers or had family support – did not highlight time constraint as much, but financial constraints were certainly echoed (medication and consultation costs). Another difference is in baseline awareness levels. Women in developed countries often have higher health literacy; for example, a study in Texas reported that over two-thirds of women with PCOS were already aware of their condition and had obtained information from healthcare providers 18. By contrast, misconceptions can be rife in South Asian contexts: an Iranian survey found some women believed that PCOS is a self-limiting problem that would resolve on its own in a few months 7. While our study did not directly quantify this, we encountered anecdotal evidence of similar misconceptions in Pakistan – e.g., some women thought that once they get married or have a child, their PCOS would “fix itself,” underscoring a gap in accurate knowledge 19. These differences in patient perceptions underscore the influence of education and counselling (or lack thereof) on adherence.

In summary, our findings not only reinforce globally observed patterns but also illuminate distinct, context-specific variations. Universally, women with PCOS struggle to adhere to complex regimens, often due to knowledge gaps, side effects, and the challenges of lifestyle change^9^, ^11^. Regionally, South Asian and Middle Eastern studies mirror our observations on familial and cultural influences, as well as resource constraints, shaping adherence behaviours 12,20. Differences between our population and higher-income settings (e.g. emphasis on family support and misconceptions versus time management issues) can be understood in light of socio-cultural and health system contexts, as discussed below. Crucially, recognizing these similarities and differences can help in devising interventions that are both informed by global best practices and tailored to local needs.

### Socio-Cultural and Healthcare Context Considerations

The Pakistani socio-cultural context provides important explanatory backdrop for some of the unique aspects of treatment adherence observed. One major factor is the family and community structure. In Pakistan, health decisions for women are often a family affair; young women may depend on parents or husbands for financial support and permission to seek treatment. This dynamic means that if the family does not fully understand PCOS or prioritize its management, the patient's adherence can suffer. Our findings of low adherence among some women can be partly attributed to such scenarios – for example, participants mentioned skipping doctor visits or visiting gynecologist a burden. Social norms also play a role: there can be a stigma attached to gynaecological conditions, and women might hide their diagnosis or downplay symptoms, reducing their engagement with care. This is somewhat different from Western contexts where individual autonomy in healthcare is higher; in our setting, social approval and support become key facilitators for adherence (as also noted in the Iranian context where family and peer support were identified as adherence facilitators) 12. Encouragingly, when families are educated and supportive, they can markedly improve a woman's ability to follow her regimen – a point that interventions should leverage.

Cultural dietary habits and lifestyle opportunities also affect adherence. Pakistani cuisine, while rich and flavourful, often includes oily and high-calorie dishes; shifting to a “bland” or restrictive diet can be demotivating as observed in our study and others 9. Moreover, opportunities for exercise, especially for women, may be limited by cultural norms around modesty and safety – for instance, women may not feel comfortable walking or jogging in public, and gyms for women might be inaccessible or costly. These socio-cultural constraints mean that adhering to lifestyle advice (the cornerstone of PCOS management) is intrinsically harder in our context than in places with more enabling environments. This could explain why lifestyle adherence in our cohort was particularly poor, and why even with knowledge of its importance, nearly 44% of Pakistani women in another study had poor lifestyle practices despite moderate knowledge levels^21^. The daily realities of our participants (busy with household responsibilities, limited recreational exercise culture, easy availability of fried foods) can override intentions to stick to diet and exercise plans.

The healthcare system in Pakistan further contextualizes our findings. Most participants were managed in public or low-cost private clinics where doctor-patient ratios are high. Consequently, as reported, consultations tend to be brief with little time for detailed counselling. This systemic issue leaves many questions unanswered for patients and can breed misconceptions (for example, 70% of women in a Canadian study reported never being informed by their doctors about PCOS's long-term risks^13^ one can imagine the communication gap might be even wider in Pakistan's resource-strapped clinics). Additionally, the healthcare system provides limited follow-up mechanisms – there are no routine reminder systems or multidisciplinary follow-ups in most cases. Once the patient leaves the clinic with a prescription and generic advice (“reduce weight, eat healthy”), there may be no check-in until the next appointment, which could be months away. During that time, without support, adherence often falters. This gap highlights why multidisciplinary support (nutritionists, counsellors, follow-up calls) is crucial, yet largely missing in our context.

Demographic factors also intersect with culture and system issues. For example, our sample included both adolescents/young adults and older reproductive-age women. Younger, unmarried women might be less adherent if they do not currently desire fertility (hence see less immediate benefit to treatment), whereas married women trying to conceive could be more motivated in the short term. Indeed, some participants hinted at using herbal supplements and homemade remedies, reflecting a pluralistic health-seeking behaviour when conventional treatments did not meet expectations. This tendency, less documented in Western literature, can lead to interruptions in evidence-based treatment.

### Implications for Clinical Practice and Public Health

The insights gained from this study have direct implications for clinical practice, health education initiatives, and public health policy in Pakistan. For clinicians and healthcare providers, a key lesson is the importance of patient-centered counselling. Doctors, gynaecologists, and endocrinologists managing PCOS should allocate time to educate patients about the nature of PCOS as a chronic condition, the expected timeline for improvements, and the critical importance of adherence. Simply prescribing medications without ensuring the patient understands why she needs to take them long-term (or why diet and exercise are vital) is insufficient and contributes to non-adherence 22. Providers should explicitly discuss common misconceptions – for example, clarifying that PCOS typically does not resolve on its own and that treatments prevent serious long-term outcomes like diabetes and endometrial hyperplasia. Additionally, healthcare providers should integrate PCOS counseling into primary care visits and strengthen patient–provider communication to improve understanding and adherence. Policy initiatives focusing on subsidized medications, community health education, and culturally tailored lifestyle programs are essential for sustainable improvement. Moreover, given the high prevalence of psychological distress, integrating mental health screening into PCOS clinics is advisable. Treating or referring mental health issues is likely to improve treatment adherence, as recommended by international PCOS guidelines 23. In practice, a clinician managing PCOS should either have access to a mental health professional or be prepared to counsel patients on stress management, thereby tackling an often “hidden” barrier to compliance.

From a health education perspective, broader community outreach is needed. Public health education campaigns can raise awareness about PCOS in the general population, which may reduce stigma and encourage family support. Community health workers and nurses can be enlisted to deliver workshops or counselling sessions about PCOS at the community level, especially in rural or low-literacy settings. Schools and colleges can also be strategic venues for dissemination, since PCOS often begins in adolescence; including PCOS awareness in reproductive health education could arm young women with knowledge before symptoms even fully manifest.

At the public health policy level, a multi-faceted strategy is required to tackle systemic barriers. First, improving access to multidisciplinary care is key. Policymakers should consider establishing specialized PCOS clinics or integrating PCOS management into existing reproductive health services at tertiary and secondary hospitals. Such clinics could offer “one-stop” services including nutrition counselling, weight management programs, and mental health support alongside medical treatment. This aligns with calls for multidisciplinary care in PCOS from patient advocacy groups in other countries 24. Secondly, incorporating PCOS into national guidelines and training for primary care providers would help standardize care. Many women in Pakistan may first present to general practitioners or lady health visitors; equipping these providers with the knowledge to counsel on PCOS (and not dismiss symptoms like irregular menses) will facilitate earlier intervention and repeated reinforcement of adherence messages. Such policy interventions would reduce the economic burden of adhering to treatment. Another policy implication is the potential use of technology: developing telehealth follow-up and counselling services can help overcome gaps in follow-up care. Regular phone calls or mobile app reminders for appointments and medication refills could significantly improve adherence, as evidenced by chronic disease management programs elsewhere. The government and private sector could collaborate on health solutions tailored for women with PCOS. In summary, policies that educate, enable, and empower patients – from clinic to community – will be essential to improve adherence and outcomes in PCOS at a population level.

### Strengths and Limitations of the Study

Our study has several strengths. To our knowledge, it is among the first to specifically investigate treatment regimen adherence in Pakistani women with PCOS, addressing an important gap in the literature. The study benefited from a decent sample size and a comprehensive approach: we not only quantified adherence levels but also explored a wide range of potential barriers (personal, treatment, and systemic) and examined key predictors like SES and BMI. We attempted to ensure diversity in our sample, recruiting participants from multiple clinics (urban and semi-urban) and including a range of ages, which improves the generalizability of the results within Pakistan.

Despite these strengths, certain limitations must be acknowledged for proper interpretation of our findings. First, the study's cross-sectional design limits our ability to draw causal inferences. We identified associations (e.g., between SES or BMI and adherence), but we cannot definitively say these factors cause better or worse adherence – longitudinal follow-up would be needed to confirm causality. Second, Participants might have overestimated their adherence due to social desirability or recall issues. Indeed, self-reported adherence tends to be higher than objective measures in many studies^25^,^11^, so the true adherence rates might be even lower than reported. Third, our sample may not fully represent all Pakistani women with PCOS. The recruitment was clinic-based, which means women who are not engaged with healthcare or who dropped out entirely would not be captured – potentially underestimating the magnitude of non-adherence in the broader PCOS population. However, these limitations do not negate the core findings but rather suggest areas for future refinement and research.

By situating these findings within the Pakistani healthcare context, this study underscores the need for multidimensional interventions that address both personal and structural barriers to adherence.

## Conclusion and Future Recommendations

This study highlights that treatment regimen adherence among women with PCOS in Pakistan is suboptimal, undermined by a confluence of personal, cultural, and healthcare-related barriers. The key findings – poor overall adherence, critical roles of knowledge gaps, treatment side effects, and support systems, and the influence of socioeconomic and BMI factors – point to the need for multifactorial interventions. To improve outcomes in this population, it is imperative that healthcare providers, community educators, and policymakers work in tandem to reduce these barriers. Clinicians should enhance patient education and follow-up, ensuring that women are not only prescribed treatments but also empowered to implement them. Public health efforts must raise awareness and destigmatize PCOS, fostering an environment where women feel supported by family and society in managing a chronic condition. On a policy level, integrating services (dietary, psychological, medical) and alleviating financial burdens will remove structural impediments to adherence.

Future research should build on these findings by exploring targeted strategies to improve adherence. Intervention studies could test the effectiveness of structured lifestyle programs tailored to Pakistani diets and living situations, or the impact of mobile health reminders and counselling on sustaining medication use and lifestyle changes. Additionally, qualitative research delving into familial and marital perspectives on PCOS treatment could inform family-centered education approaches. Longitudinal studies are recommended to track adherence patterns over time – for instance, following newly diagnosed patients through the first year of treatment – to identify critical drop-off points and reasons. Such research would also clarify the long-term health impacts of improved adherence (e.g., reductions in weight, prevention of diabetes) in our context, bolstering the case for investing in adherence interventions. Ultimately, improving treatment adherence in PCOS is a vital step toward mitigating the long-term sequelae of this syndrome in Pakistan. By implementing culturally informed, evidence-based strategies, we can enhance the quality of life and health outcomes for thousands of women affected by PCOS, translating clinical research into tangible public health benefit.

## Figures and Tables

**Table 1:** Demographic variables of subjects (n=401)

Variables	f	%
**Age (years)**		
13-20	84	20.9
21-28	194	48.4
29-36	81	20.2
36-45	42	10.5
**Marital Status**		
Single	232	57.9
Married	129	32.2
Divorced	27	6.7
Widow	13	3.2
**Education**		
Primary School	35	8.7
High school	95	23.7
Undergraduate	180	44.9
Postgraduate	91	22.7
**Socioeconomic Status**		
Upper Class	126	31.4
Upper Middle Class	130	32.4
Lower-Middle Class	114	28.4
Lower Class	31	7.7
**Occupation**		
Student	35	8.7
Employed	116	28.9
Homemaker	193	48.1
Unemployed	57	14.2
**Residence**		
Urban	220	54.9
Semi Urban	96	23.9
Rural	68	17.0
Remote Areas	17	4.2
**BMI**		
Underweight	40	10.0
Normal	175	42.6
Overweight	148	37.2
Obese	38	10.2

**Table 2:** Prescribed treatment strategies for women with PCOS (n=401)

Treatment profile	f	%
**Medication**		
Oral Contraceptive pills	103	25.7
Metformin	131	32.7
Anti-Androgens	81	20.2
Others (Hormonal Therapy)	86	21.4
**Physical Activity**		
Brisk Walking	171	42.6
Jogging	69	17.2
Yoga	42	10.5
Sedentary Lifestyle	119	29.7
**Diet**		
Whole Wheat chapati, Bread, rice	115	28.7
Fruits, Vegetables, lentils	84	20.9
Junk Foods	152	37.9
Meat (Fish, Mutton, Chicken, Beef)	50	12.5
**Lifestyle Modifications**		
Diet Changes	113	28.2
Exercise	128	31.9
Stress Management	98	24.4
None	62	15.5
**Complementary Therapies**		
Herbal Remedies	77	19.2
Homeopathic Treatment	66	16.5
Only Prescribed Treatment	160	39.9
Home Remedies	98	24.4

**Table:** Adherence to Treatment Regimen

Level of Adherence	f	%
**Adherent**	**86**	**21.4**
**Partially Adherent**	**203**	**50.6**
**Non-Adherent**	**112**	**27.9**

**Figure 1:**
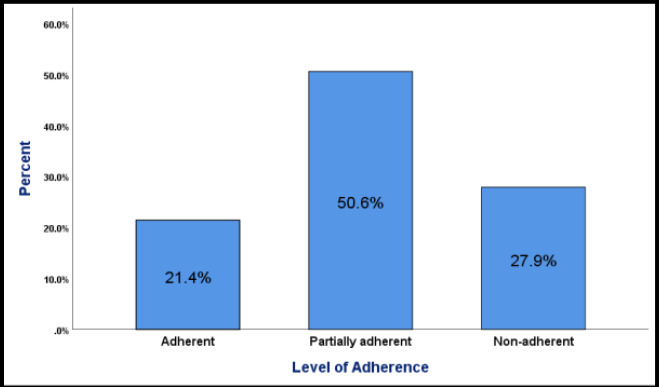
Bar Chait representing the level of adherence among participants

**Table 4:** Distribution of the barriers to treatment adherence (n=401)

Barriers	f	%
**Personal Factors**		
Knowledge	160	39.9
Fear of Infertility	233	58.1
Visiting a gynaecologist was a burden	176	43.9
Cultural or social taboos	160	39.9
Craving for restricted food	186	46.4
Lack of motivation from family or peer	170	42.4
Lack of time for exercise	266	66.3
Physical exercise tiring	235	58.6
**Treatment-Related Factors**		
Cost of the drug	167	41.6
Long duration of therapy	250	62.3
No relief of symptoms	183	45.6
Bland diet	160	39.9
Side effects	218	54.4
**Health Care System Related Factors**		
Long-distance from hospital	184	45.9
Lack of informational support from the doctor	160	39.9
Lack of privacy during consultation	145	36.2
Lack of proper communication by doctor	159	39.7
Difficulty in getting appointments	169	42.1
Poor access to medications	135	33.7

**Table 5:** Association Between Sociodemographic Variables and Adherence Using Chi-square Test (N=401)

Variables	f	%	X^2^	df	p-value
**Age (years)**					
13-20	84	20.9	2.124	3	0.547
21-28	194	48.4			
29-36	21	20.2			
36-45	42	10.5			
**Marital Status**					
Single	232	57.9	0.916	3	0.822
Married	129	32.2			
Divorced	27	6.7			
Widow	13	3.2			
**Education**					
Primary School	35	8.7	2.995	3	0.392
High school	95	23.7			
Undergraduate	180	44.9			
Postgraduate	91	22.7			
**Socioeconomic Status**					
Upper Class	126	31.4	8.980	3	**0.030**
Upper Middle Class	130	32.4			
Lower-Middle Class	114	28.4			
Lower Class	31	7.7			
**Occupation**					
Student	35	8.7	2.091	3	0.554
Employed	116	28.9			
Homemaker	193	48.1			
Unemployed	57	14.2			
**Residence**					
Urban	220	54.9	5.623	3	0.131
Semi Urban	96	23.9			
Rural	68	17.0			
Remote Areas	17	4.2			
**BMI**					
Underweight	40	10.0	8.278	3	**0.041**
Normal	175	42.6			
Overweight	148	37.2			
Obese	38	10.2			

**Table 6:** Binary logistic regression analysis of predictors of treatment adherence among women with PCOS (N = 401)

95% Wald Confidence Interval for Exp(B)				
Sociodemographic Characteristics	Exp (B)	Lower	Upper	Sig.
**Age Group**				0.398
13 -20	1.197	0.443	3.238	0.723
21 -28	1.709	1.709	3.825	0.192
29 -36	1.692	1.692	3.949	0.224
36- 45	**Reference Group**			
**Marital Status**				0.843
Single	**Reference Group**			
Married	0.833	0.483	1.437	0.511
Divorced	1.008	0.411	2.467	0.987
Widow	1.383	0.393	4.866	0.613
**Education**				0.792
Primary School	1.325	0.535	3.279	0.543
High School	1.392	0.690	2.809	0.356
Undergraduate	1.095	0.614	1.950	0.759
Postgraduate	**Reference Group**			
**Occupation**				0.716
Student	1.131	0.446	2.867	0.796
Employed	0.766	0.369	1.593	0.476
Homemaker	0.794	0.413	1.528	0.490
Unemployed	**Reference Group**			
**Socioeconomic Status**				**0.035**
Upper class	1.115	0.328	0.918	0.022
Upper middle class	0.649	0.285	1.479	0.304
Lower middle class	0.549	0.666	1.865	0.680
Lower Class	**Reference Group**			
**Residence**				0.141
Urban	**Reference Group**			
Semi-Urban	1.222	0.730	2.044	0.445
Rural	1.404	0.781	2.524	0.257
Remote Areas	0.296	0.078	1.120	0.073
**Body Mass Index (B MI)**				**0.048**
Underweight	1.394	0.717	4.488	0.212
Normal	1.467	0.704	3.056	0.048
Overweight	0.831	0.391	1.764	0.630
Obese	**Reference Group**			

(Note: Reference categories for comparison—Age Group: 36+ years, Marital Status: Single, Education: Postgraduate, Occupation: Unemployed, Socioeconomic Status: Lower class, Residence: Urban, BMI: Obese.)

## References

[1] Vijayan SM, Kalaivani H, Mitra S, John J, Anila A, Boban L (2022). Barriers to treatment regimen adherence in Indian women with polycystic ovarian syndrome. Journal of Family Medicine and Primary Care.

[2] Butt MS, Saleem J, Zakar R, Aiman S, Bukhari GMJ, Fischer F (2024). Comparison of physical activity levels and dietary habits between women with polycystic ovarian syndrome and healthy controls of reproductive age: a case-control study. BMC Women's Health.

[3] Yu JH, Moon MK, Ahn HC, Yang Y-M (2024). Assessing medication use patterns among patients with polycystic ovary syndrome at a tertiary care teaching hospital in South Korea: A retrospective study. Medicine.

[4] Bai H, Ding H, Wang M (2024). Polycystic Ovary Syndrome (PCOS): Symptoms, Causes, and Treatment. CEOG.

[5] Teede HJ, Misso ML, Costello MF, Dokras A, Laven J, Moran L (2018). Recommendations from the international evidence-based guideline for the assessment and management of polycystic ovary syndrome. Human Reproduction.

[6] Pirotta S, Joham AE, Moran LJ, Skouteris H, Lim SS (2021). Implementation of the polycystic ovary syndrome guidelines: A mixed method study to inform the design and delivery of a lifestyle management program for women with polycystic ovary syndrome. Nutrition & Dietetics.

[7] Bazarganipour F, Taghavi SA, Allan H, Hosseini N (2017). Facilitating and inhibiting factors related to treatment adherence in women with polycystic ovary syndrome: A qualitative study. International Journal of Reproductive Biomedicine.

[8] Naz S, Anjum N, Gul I (2020). A community based cross sectional study on prevalence of polycystic ovarian syndrome (PCOS) and health related quality of life in Pakistani females.

[9] Vijayan SM, Kalaivani H, Mitra S, John J, Anila A, Damini (2022). Barriers to treatment regimen adherence in Indian women with polycystic ovarian syndrome. Journal of Family Medicine and Primary Care.

[10] Sacre H, Haddad C, Hajj A, Zeenny RM, Akel M, Salameh P (2023). Development and validation of the socioeconomic status composite scale (SES-C). BMC Public Health.

[11] Parker M, Warren A, Nair S, Barnard M (2020). Adherence to treatment for polycystic ovarian syndrome: A systematic review. PLoS One.

[12] Ameli N, Brown M (2023). A Qualitative Study of Barriers and Facilitators to Polycystic Ovary Syndrome Treatment Adherence: Iranian Context. J Comm Med and Pub Health Rep.

[13] Sydora BC, Wilke MS, McPherson M, Chambers S, Ghosh M, Vine DF (2023). Challenges in diagnosis and health care in polycystic ovary syndrome in Canada: a patient view to improve health care. BMC Women's Health.

[14] Li S, He A, Yang J, Yin T, Xu W (2011). A logistic regression analysis of factors related to the treatment compliance of infertile patients with polycystic ovary syndrome. The Journal of Reproductive Medicine.

[15] Ganie MA, Rashid A, Sahu D, Nisar S, Wani IA, Khan J (2020). Prevalence of polycystic ovary syndrome (PCOS) among reproductive age women from Kashmir valley: A cross-sectional study. International Journal of Gynecology & Obstetrics.

[16] Ko H, Teede H, Moran L (2016). Analysis of the barriers and enablers to implementing lifestyle management practices for women with PCOS in Singapore. BMC Research Notes.

[17] Arasu A, Moran LJ, Robinson T, Boyle J, Lim S (2019). Barriers and facilitators to weight and lifestyle management in women with polycystic ovary syndrome: general practitioners' perspectives. Nutrients.

[18] Rao M, Broughton KS, LeMieux MJ (2020). Cross-sectional study on the knowledge and prevalence of PCOS at a multiethnic university. Progress in Preventive Medicine.

[19] Athar Z, Javed N (2024). Exploring the Lived Experiences and Coping Mechanisms of Unmarried Women with Polycystic Ovary Syndrome (PCOS) in Pakistan. Sociological Research And Innovation.

[20] Aladin M (2024). A Cultural Perspective on the Lived Experience of South Asian American Women With PCOS.

[21] Nadeem N, Zerish Z, Niazi SG, Sahi AA, Babar A, Khalid R (2024). Knowledge, Aptitude, and Practice Study Regarding Modifiable Factors Among Females Diagnosed With PCOS: KAP on Modifiable Factors in PCOS. Journal of Health and Rehabilitation Research.

[22] Rashid R, Geer M, Verma S, Rashid F, Ganaie A (2019). Evaluation of prescription pattern and compliance in kashmiri women with polycystic ovarian syndrome. Journal of Drug Delivery and Therapeutics.

[23] Teede HJ, Tay CT, Laven JJ, Dokras A, Moran LJ, Piltonen TT (2023). Recommendations from the 2023 international evidence-based guideline for the assessment and management of polycystic ovary syndrome. European Journal of Endocrinology.

[24] Tay CT, Pirotta S, Teede HJ, Moran LJ, Robinson T, Skouteris H (2021). Polycystic ovary syndrome models of care: a review and qualitative evaluation of a guideline-recommended integrated care. Seminars in Reproductive Medicine.

[25] Lin AW, Kazemi M, Jarrett BY, Vanden Brink H, Hoeger KM, Spandorfer SD (2019). Dietary and physical activity behaviors in women with polycystic ovary syndrome per the new international evidence-based guideline. Nutrients.

